# Signal processing in urodynamics: towards high definition urethral pressure profilometry

**DOI:** 10.1186/s12938-016-0145-6

**Published:** 2016-03-22

**Authors:** Mario Klünder, Oliver Sawodny, Bastian Amend, Michael Ederer, Alexandra Kelp, Karl-Dietrich Sievert, Arnulf Stenzl, Ronny Feuer

**Affiliations:** Institute for System Dynamics, University of Stuttgart, Waldburgstr. 17/19, 70563 Stuttgart, Germany; Department of Urology, University of Tübingen, Hoppe-Seyler-Straße 3, 72076 Tübingen, Germany; Department of Urology, Paracelsus Medical University of Salzburg, Müllner Hauptstr. 48, 5020 Salzburg, Austria

**Keywords:** Signal processing, Urodynamics, Urethral pressure

## Abstract

**Background:**

Urethral pressure profilometry (UPP) is used in the diagnosis of stress urinary incontinence (SUI) which is a significant medical, social, and economic problem. Low spatial pressure resolution, common occurrence of artifacts, and uncertainties in data location limit the diagnostic value of UPP. To overcome these limitations, high definition urethral pressure profilometry (HD-UPP) combining enhanced UPP hardware and signal processing algorithms has been developed. In this work, we present the different signal processing steps in HD-UPP and show experimental results from female minipigs.

**Methods:**

We use a special microtip catheter with high angular pressure resolution and an integrated inclination sensor. Signals from the catheter are filtered and time-correlated artifacts removed. A signal reconstruction algorithm processes pressure data into a detailed pressure image on the urethra’s inside. Finally, the pressure distribution on the urethra’s outside is calculated through deconvolution. A mathematical model of the urethra is contained in a point-spread-function (PSF) which is identified depending on geometric and material properties of the urethra. We additionally investigate the PSF’s frequency response to determine the relevant frequency band for pressure information on the urinary sphincter.

**Results:**

Experimental pressure data are spatially located and processed into high resolution pressure images. Artifacts are successfully removed from data without blurring other details. The pressure distribution on the urethra’s outside is reconstructed and compared to the one on the inside. Finally, the pressure images are mapped onto the urethral geometry calculated from inclination and position data to provide an integrated image of pressure distribution, anatomical shape, and location.

**Conclusions:**

With its advanced sensing capabilities, the novel microtip catheter collects an unprecedented amount of urethral pressure data. Through sequential signal processing steps, physicians are provided with detailed information on the pressure distribution in and around the urethra. Therefore, HD-UPP overcomes many current limitations of conventional UPP and offers the opportunity to evaluate urethral structures, especially the sphincter, in context of the correct anatomical location. This could enable the development of focal therapy approaches in the treatment of SUI.

**Electronic supplementary material:**

The online version of this article (doi:10.1186/s12938-016-0145-6) contains supplementary material, which is available to authorized users.

## Background

Urethral pressure profilometry (UPP) is used in the diagnosis of stress urinary incontinence (SUI). SUI is a significant medical, social and economic problem, as about 15 % of all females and 10 % of all males are affected in western countries. Among the elderly even more than 25 % are affected by SUI [1]. In order to perform UPP, a measurement catheter is pulled slowly, by a defined distance/time through the urethra thus obtaining a pressure profile along the inside of the urethra. Four catheter types are common in clinical use today: water-filled infused catheters, water-filled latex balloons, air-charged balloon catheters, and microtip catheters [2, 3]; with each type possessing certain advantages and disadvantages compared to the others.

Only water-filled systems can compensate for hydrostatic pressure caused by different vertical sensor positions of the vesical pressure sensor [2]. On the other hand, they are prone to artifacts due to kinks or air bubbles in the tube, and their reference level needs to be reset when the patient changes position [3]. This led to microtip catheters becoming the benchmark technology in multichannel urodynamics due to the reproducibility and reliability of their data [4–6]. However, as they are reusable they are comparatively expensive and require extensive cleaning and sterilization [5]. Furthermore, they are considered prone to artifacts as they measure pressure in a unidirectional fashion [7]. Therefore, air-charged balloon-type catheters have gained popularity as they are single use (avoiding re-sterilization or possible transmission of infections) and easy to use. They are considered to deliver excellent reproducibility reliability for UPP measurement even better than microtip catheters, as they measure pressure circumferentially which is considered more accurate and less prone to artifacts than unidirectional measurements [3, 6]. Compressibility of air, however, may negatively affect pressure recording fidelity [8] and the size of the balloon decreases resolution along the urethra as it acts as a moving-average filter.

Additionally, angular fluctuations in urethral pressure profiles have been observed (e.g. [7, 9]). In order to assess these, balloon type catheters cannot be used. Microtip catheters can simultaneously record pressure data at different angular orientations while retaining a small diameter due to the small size of their pressure sensors. The cause of angular pressure fluctuations is still unclear. Rossier et al. [9] observed higher pressures at the 6 o’clock position than at the 12 o’clock position with back-to-back mounted sensors. After sphincterotomy or pudendal block this difference was annulled; they therefore conclude that the cause of the discrepancy is due to circumferential deformation and change in urethral wall tension. Griffiths [7] on the other hand states that normal stress around the urethra must be in a static equilibrium in any given cross section, else they are artifacts caused by catheter bending. Lose et al. [10] conclude that the measurement of urethral pressures currently cannot “(1) discriminate urethral incompetence from other disorders; (2) provide a measure of the severity of the condition; (3) provide a reliable indicator to surgical success.” Therefore, the diagnostic value of UPP for the diagnosis of incontinence seems to be limited.

In order to overcome these shortcomings we propose a new approach called high definition urethral pressure profilometry (HD-UPP). Its goals are (1) to significantly increase spatial resolution compared to systems in clinical use today; (2) to eliminate artifacts; (3) to reconstruct pressure information in gaps between sensors; (4) to reconstruct the pressure distribution on the urethra’s outside thus assessing sphincter strength distribution. Preliminary results were reported in our conference papers [11–13] and in [14]. In this publication, all processing steps in HD-UPP are coherently presented for the first time. We briefly summarize the methods presented in previous papers and extend the model for calculating the pressure distribution on the urethra’s outside from one parameter set to a range of model parameters. Additionally, the model’s pressure transmission properties are analyzed and exploited in the frequency domain. We apply the proposed signal processing steps to experimental data from female minipigs. The results are shown and differences between pressure images inside and outside the urethra are discussed.

## Methods

### HD-UPP hardware

**Fig. 1:**
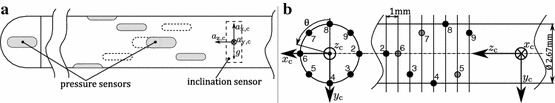
HD-UPP catheter prototype. Prototype of a novel microtip catheter with nine pressure sensors and an inclination sensor (**a**) [11]. Sensor configuration on the microtip catheter (**b**). Sensor #1 is located at the tip as shown in (**a**)

We used a novel custom-built HD-UPP catheter prototype (UNISENSOR AG, Attikon, Switzerland) with a total of nine pressure sensors, one at the tip, the others equally distributed around the circumference 6 cm from the tip (Fig. 1a). The sensor configuration is shown in detail in Fig. 1b. All $n_{\mathrm {S}}=8$ sensors measuring the pressure inside the urethra are placed at $45^\circ$ intervals around the circumference. The numbers denote the sensor number; note that sensor #1 is the sensor at the tip which only measures the reference pressure inside the urinary bladder. The remaining sensors are placed at 1 mm intervals in axial direction with sensor #2 being closest to the tip. We use an eight French catheter which corresponds to a diameter of about 2.7 mm.

**Fig. 2:**
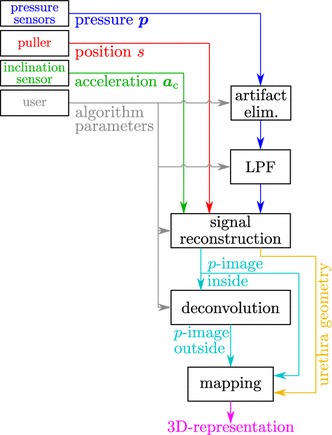
Signal flow in HD-UPP. Pressure data $\varvec{p}$ are preprocessed through artifact elimination and a lowpass filter (LPF). Together with position and acceleration data a pressure image on the urethra’s inside is reconstructed and the urethral geometry in the sagittal plane calculated. The pressure image on the urethra’s outside is obtained through deconvolution and the results are mapped onto a 3D-representation of the urethra. User defined parameters control the algorithms’ behavior such as resolution of the pressure image

**Fig. 3:**
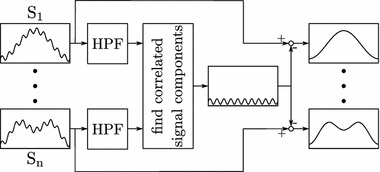
Basic concept for removing time-correlated artifacts. Sensor signals S_1_ to S_n_ are highpass filtered (block HPF). Then, correlated signal component are extracted. Finally, those components are subtracted from the original signals

**Fig. 4:**
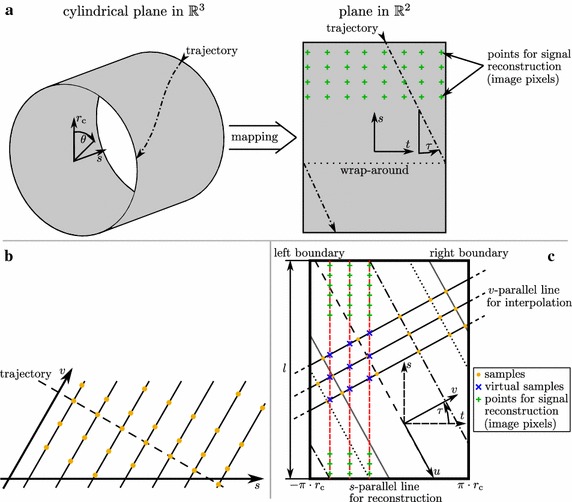
Sampling lattice in HD-UPP. Sensor trajectories (*dash-dotted line*) lie on a cylindrical surface in $\mathbb {R}^3$. For easier calculations and graphical representation this surface is mapped onto a plan in $\mathbb {R}^2$. Points for signal reconstruction (*green* +) are placed on that surface in a regular grid (**a**) [14]. Butzer and Hinsen sampling lattice with a skewed coordinate system (**b**). Coordinate system and sampling lattice for signal reconstruction in HD-UPP (**c**) [11]. *Lines* parallel to *u* represent different sensor trajectories

The microtip catheter was connected to a signal amplifier specifically designed for this device (UNISENSOR AG, Attikon, Switzerland). We used a linear motor (DSZY1-24-30-200, Drive-System Europe Ltd., Werther, Germany) with an integrated position sensor as a puller device. All sensors and actuators were controlled through a dSPACE 1103 system (dSPACE GmbH, Paderborn, Germany) which used a sampling rate of $f_{\mathrm {s}}$ = 1 kHz.

An integrated triaxial acceleration sensor (BMA250, Bosch Sensortec, Reutlingen, Germany) was used to determine the orientation of the catheter during the measurement relative to earth’s gravity field. Data on the catheter’s orientation are needed to spatially locate pressure data for signal processing. As the catheter was pulled with a constant velocity, we assume other acceleration effects to be negligible compared to gravity. The rotational orientation is determined by the angle $\theta$, which is considered to be $0^\circ$ when the sensor’s $y_{\mathrm {c}}$-axis is aligned with earth’s gravity field. Combining data from the inclination sensor with position data from the puller device enables spatial location of each sample from the pressure sensors. This catheter has been evaluated and compared to an air-charged system, delivering plausible results in agreement with previously conducted comparisons of microtip and air-charged systems [14].

It is often pointed out that microtip catheters do not measure hydrostatic pressure, as hydrostatic pressure is a scalar quantity whereas data from microtip catheters are directional and therefore an area force or normal stress on the sensor’s surface from the urethral tissue (e.g. [7, 15, 16]). However, for the sake of readability, we will refer to data from the microtip catheter as “pressure” throughout this paper, keeping its directional properties in mind.

### Signal processing steps

Preliminarily, a few coordinate systems necessary in the signal processing steps need to be defined. The catheter’s local coordinate system is shown in Fig. 1b. $z_{\mathrm {c}}$ is pointing along the catheter’s axis towards the tip. $x_{\mathrm {c}}$ points orthogonally to $z_{\mathrm {c}}$ towards sensor #6 and $y_{\mathrm {c}}$ is added as to yield a right-hand orthonormal coordinate system. For the urethra, a curved cylindrical coordinate system is employed. The coordinate along the urethra is *s*, its radial coordinate is *r* and its angular coordinate is $\theta$. Due to the usually very slight curvature of the urethra in female minipigs, it is assumed in most signal processing steps that the urethra is straight. Finally, there is the Cartesian body coordinate system *x*, *y*, *z*. The *xz*-plane is the sagittal plane and the *yz*-plane is the horizontal plane. We assume throughout this work, that the urethra lies in the sagittal plane, therefore, the *s*-coordinate lies within the *xz*-plane.

The main signal processing steps are shown as a block-diagram in Fig. 2. Data passed to the signal processing algorithms from HD-UPP hardware include acceleration data $\varvec{a}_{\mathrm {c}}$, puller position *s* and pressure data $\varvec{p}$ from the eight urethral pressure sensors. Additionally, there are more than 60 user-defined parameters controlling the behavior of the algorithms, such as resolution of the pressure image, artifact elimination mode, model properties of the urethra, and what data to plot.

**Fig. 5:**
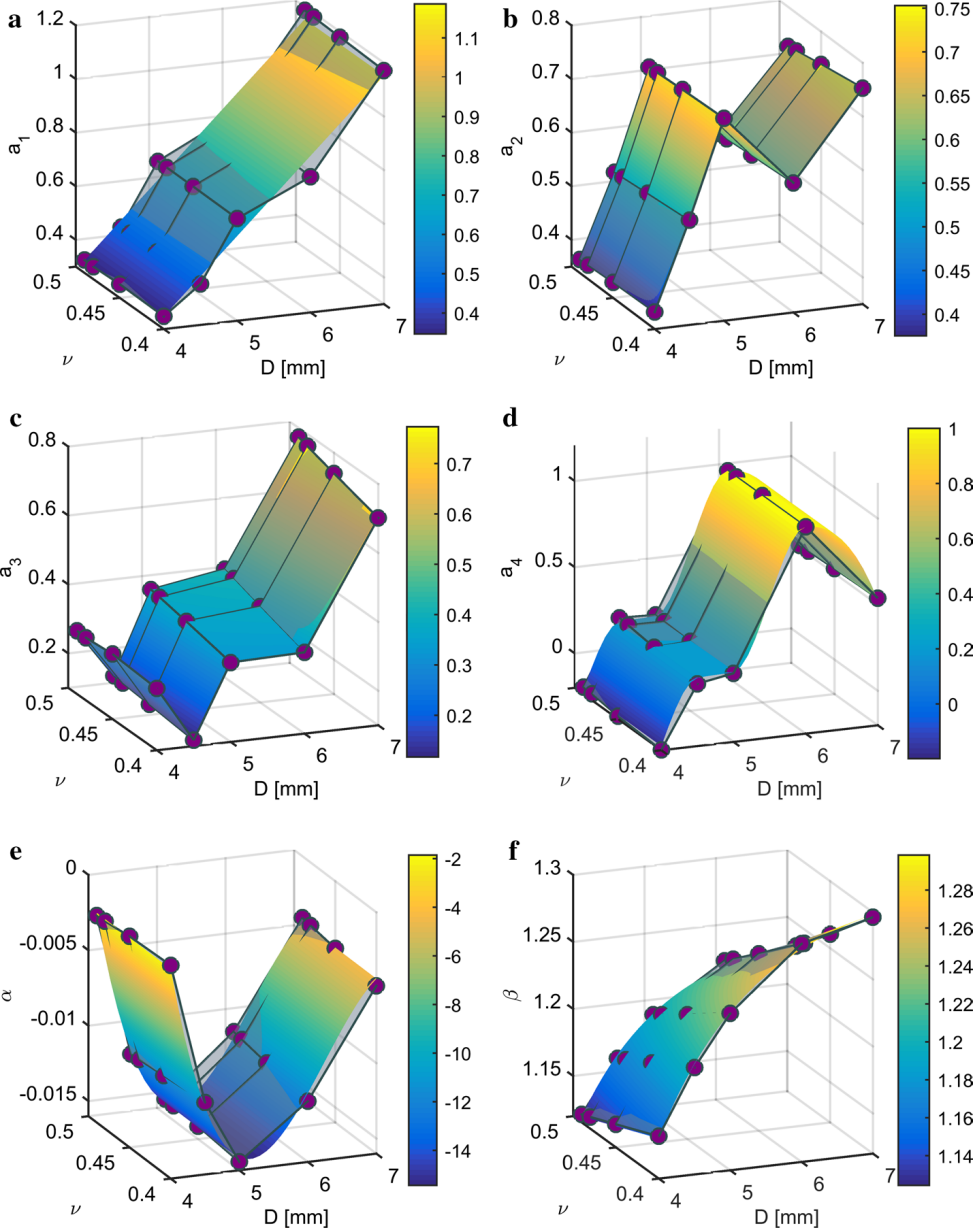
Identified PSF shape parameters. Identified shape parameters (*gray* transparent surface connecting *purple dots*) and interpolating function (*colored surface*)

**Fig. 6:**
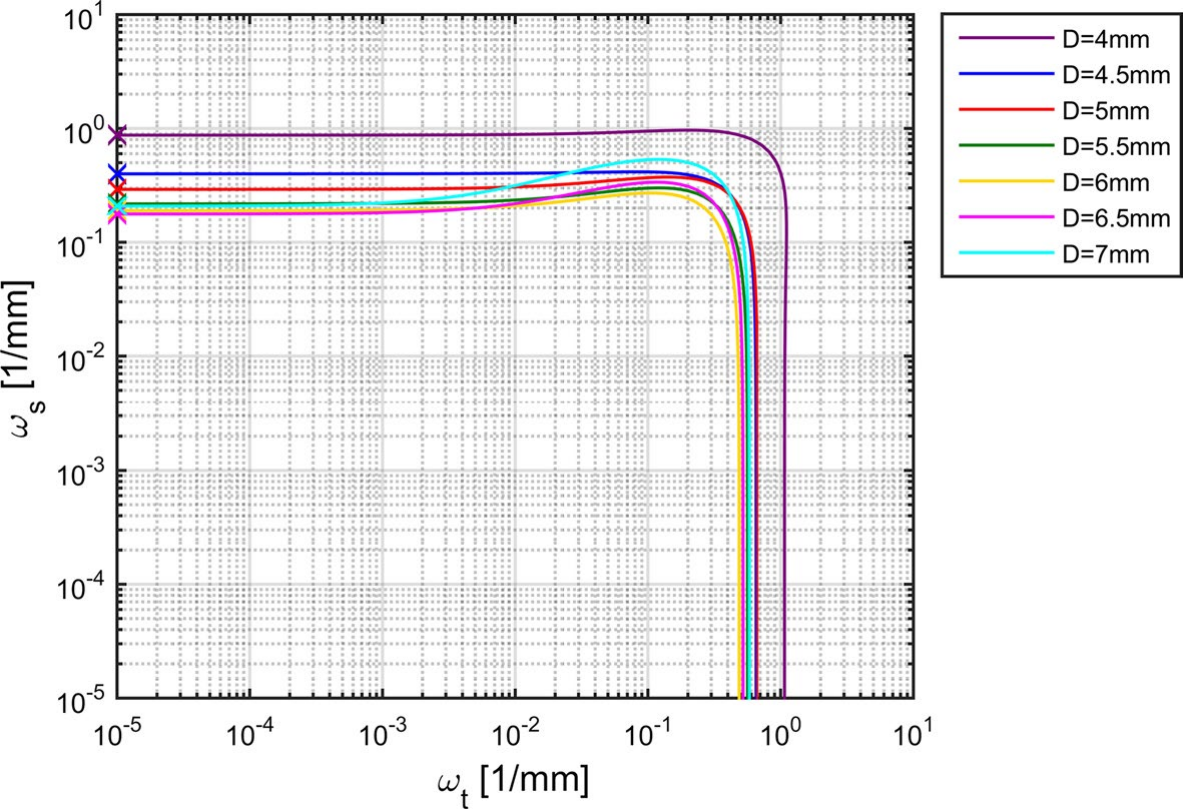
Cutoff-frequencies of identified PSFs. The *colored lines* show the PSFs’ $-6\; {\mathrm{dB}}$ cutoff-frequencies for different *D*.* x* marks the location of the lowest value on each line

**Fig. 7:**
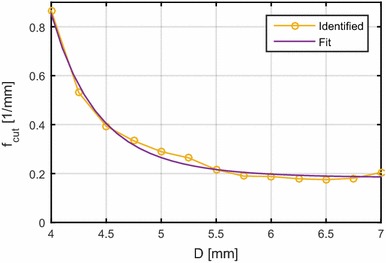
Minimal cutoff-frequencies. Identified minimal $-6\; {\mathrm{dB}}$ cutoff-frequencies and fitted function

**Fig. 8:**
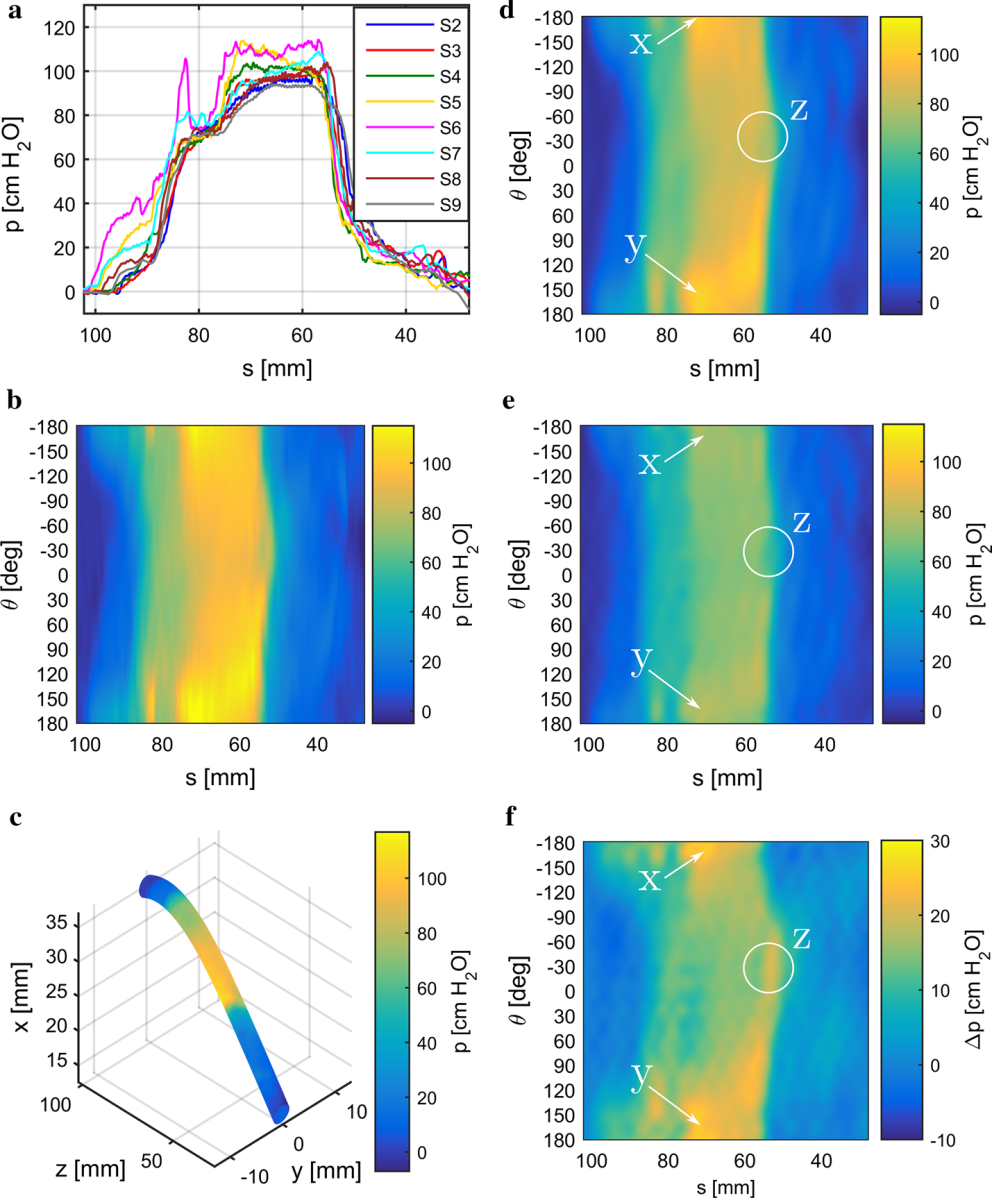
Results for minipig 1. Pressure traces with the cutoff-frequency limited by signal reconstruction (**a**). Reconstructed pressure images inside (**b**) reconstructed from pressure traces in (**a**). Pressure image inside from (**b**) mapped onto urethra geometry in the sagittal plane (*xz*) (**c**). *yz* represents the horizontal plane. Reconstructed pressure image inside with the cutoff-frequency limited by the PSF for $D=7\,{\mathrm{mm}}$ and $\nu = 0.48$ (**d**). Deconvolved pressure image (**e**) based on (**d**). Difference between (**d**) and (**e**) shown in (**f**)

The first step is to remove time-correlated artifacts from the pressure data (block artifact elim.). Next, the pressure traces are lowpass filtered (block LPF). All data are passed on to the signal reconstruction algorithm, which calculates the pressure distribution (or pressure image) on the urethra’s inside. Additionally, the urethra’s geometry in the sagittal plane is obtained. The pressure image is passed on to the deconvolution algorithm, which calculates the pressure distribution at a defined depth inside the tissue. Finally, both pressure images can be mapped onto the urethra’s geometry to obtain a 3D-representation of the pressure distribution in and around the urethra.

All subsequently described signal processing steps were carried out in Matlab^®^ R2015a.

#### Artifact elimination

Urethral pressure profiles are prone to artifacts which can be attributed to various sources. While some artifacts like pressure spikes due to coughing can be easily compensated by subtracting vesical from urethral pressure, others like vascular pulsation [17, 18] cannot be compensated for that way. In order to effectively eliminate most artifacts without loosing details in the pressure signals, we take advantage of the catheter’s high sensor count and their axial gaps between one another (Fig. 1b). Most artifacts will register at the same time at each sensor; i.e. they are correlated in the time domain across the signals whereas anatomical signal components are correlated in the space domain, that is after the axial sensor offsets have been compensated. Additionally, artifacts and physiological signal components occur mostly in separate frequency bands which is also taken into account.

**Fig. 9:**
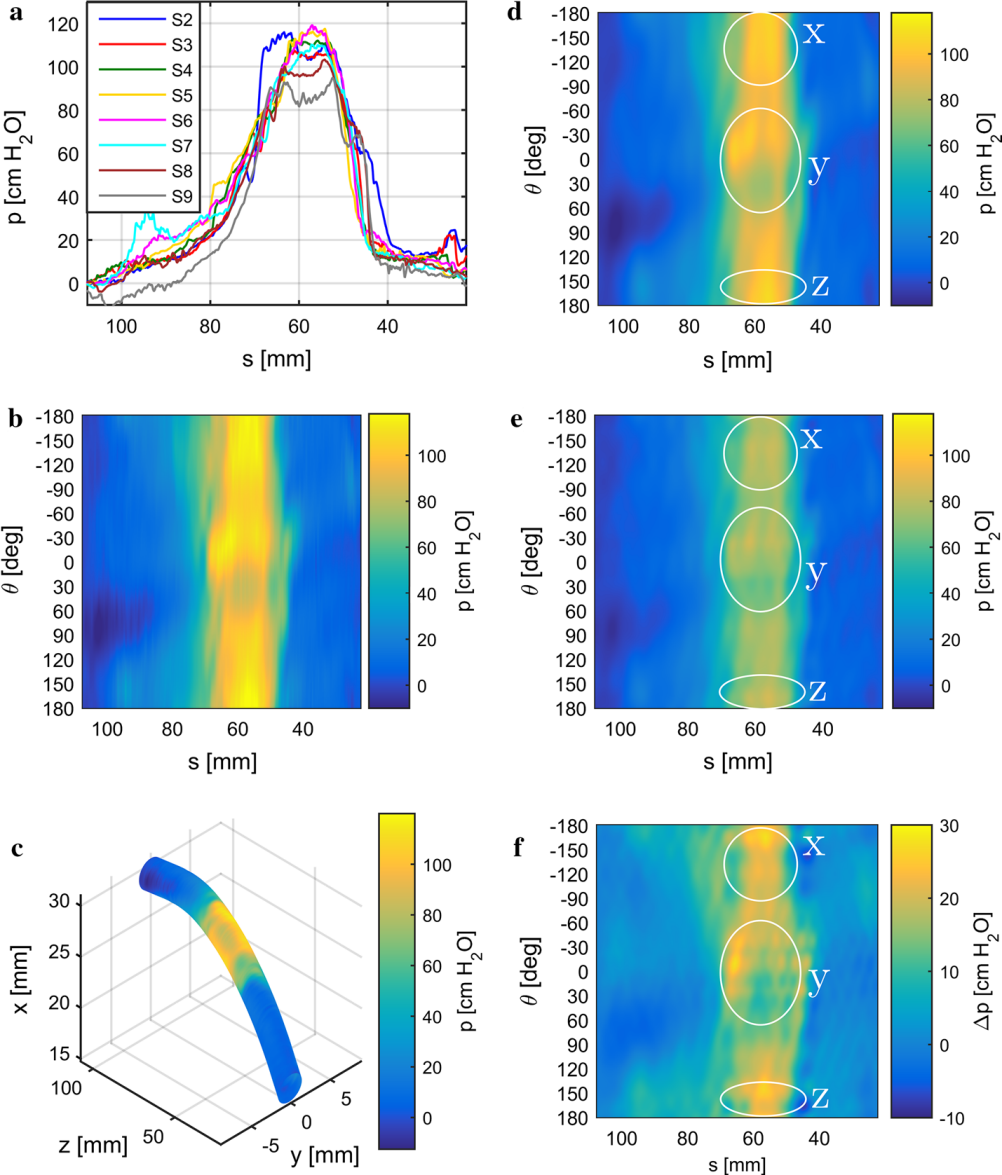
Results for minipig 2. Pressure traces with the cutoff-frequency limited by signal reconstruction (**a**). Reconstructed pressure images inside (**b**) reconstructed from pressure traces in (**a**). Pressure image inside from (**b**) mapped onto urethra geometry in the sagittal plane (*xz*) (**c**). *yz* represents the horizontal plane. Reconstructed pressure image inside with the cutoff-frequency limited by the PSF for $D= 7\,{\mathrm{mm}}$ and $\nu = 0.48$ (**d**). Deconvolved pressure image (**e**) based on (**d**). Difference between (**d**) and (**e**) shown in (**f**)

**Fig. 10:**
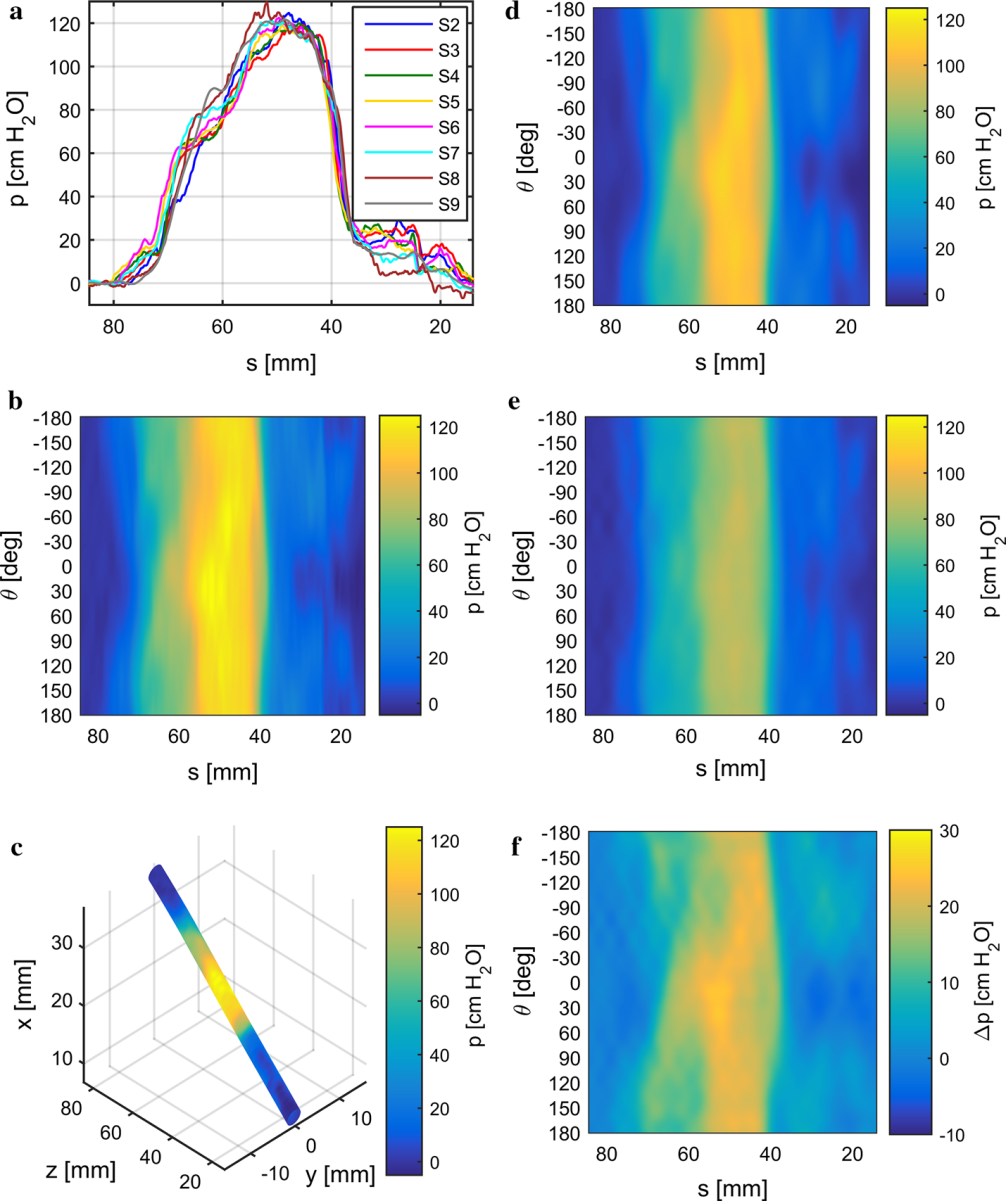
Results for minipig 3. Pressure traces with the cutoff-frequency limited by signal reconstruction (**a**). Reconstructed pressure images inside (**b**) reconstructed from pressure traces in (**a**). Pressure image inside from (**b**) mapped onto urethra geometry in the sagittal plane (*xz*) (**c**). *yz* represents the horizontal plane. Reconstructed pressure image inside with the cutoff-frequency limited by the PSF for $D= 7\,{\mathrm{mm}}$ and $\nu = 0.48$ (**d**). Deconvolved pressure image (**e**) based on (**d**). Difference between (**d**) and (**e**) shown in (**f**)

**Fig. 11:**
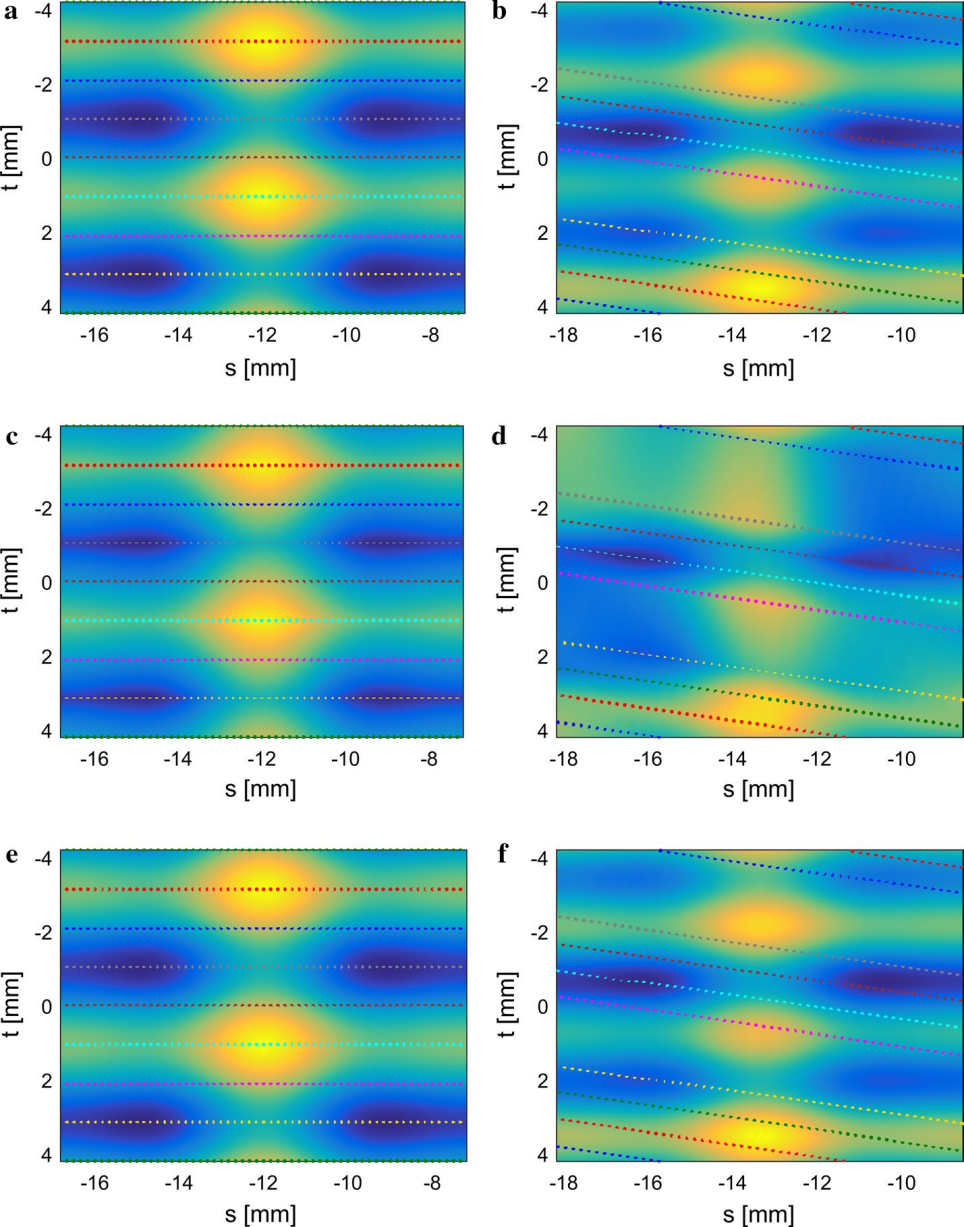
Signal reconstruction vs. bilinear interpolation. *Colored dots* represent samples from different pressure sensors. *Left column* Test function a, straight retraction. *Right column* Test function b, retraction with constant catheter rotation. *Top row* Original, sampled function. *Middle row* Delaunay-triangulation and bilinear interpolation. *Bottom row* Signal reconstruction from samples

**Fig. 12:**
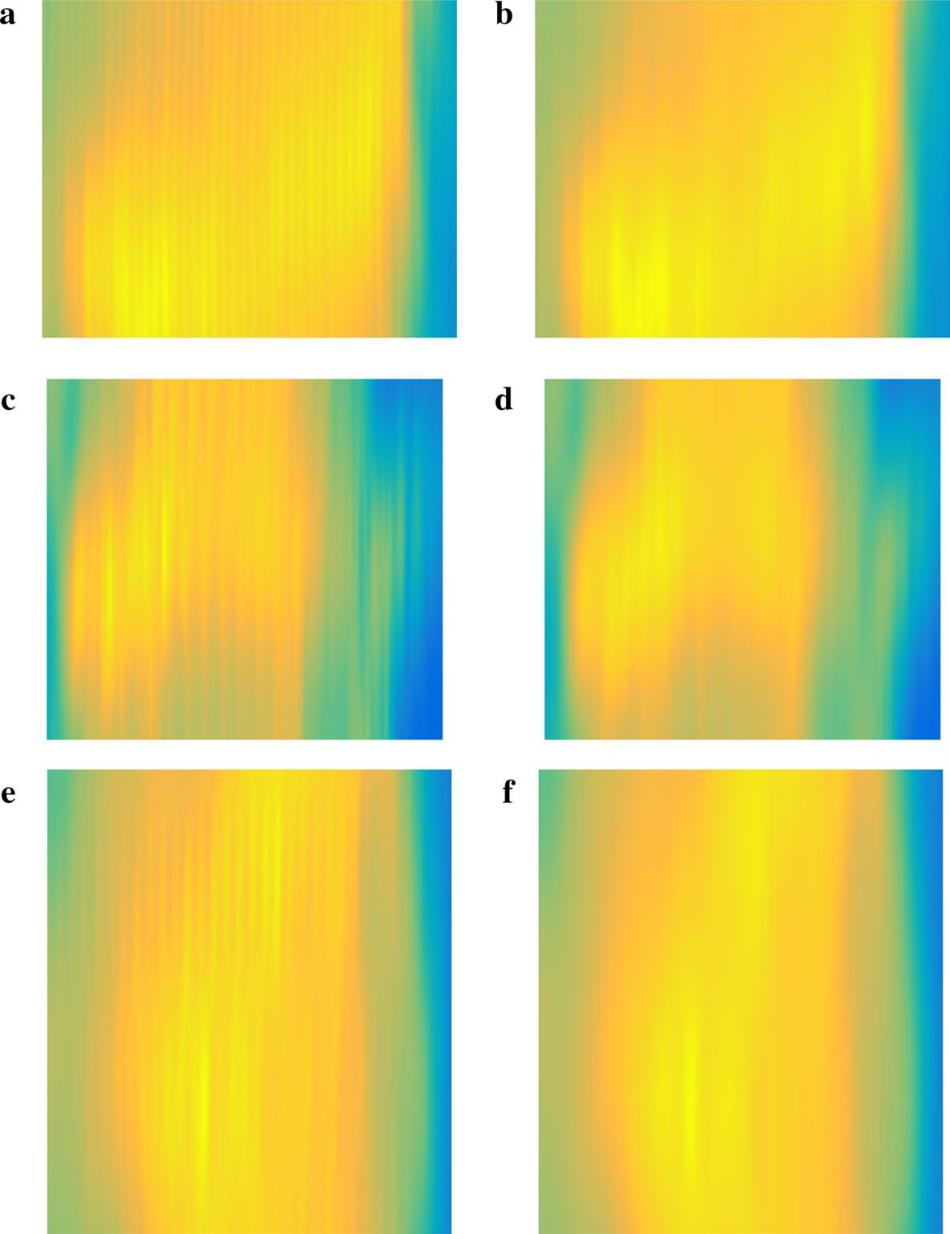
Results for artifact elimination. Rows from *top to bottom* Minipigs 1–3, respectively. *Left column* Cropped pressure images inside without artifact elimination. *Right column* Cropped pressure images inside with artifact elimination

Two methods employing this knowledge have been developed and successfully tested [13]. The basic concept for removing time-correlated artifacts is shown as a block diagram in Fig. 3. Time-correlated artifact elimination through an input model calculates the common signal component to all pressure signals. The common input is then subtracted from all pressure signals. Principal component analysis decorrelation (PCAD) decomposes the pressure signals into their (uncorrelated) principal components. Artifacts are mostly contained in the first one or two components with the highest variance, which are subtracted from the original signals to remove them. Both methods are effective at removing time-correlated artifacts from urethral pressure data while preserving details. The input model is easy to implement for online artifact estimation and canceling. PCAD is slightly more effective at removing artifacts and can be adjusted in its intensity through the number of subtracted components.

#### Lowpass filtering

The high sampling rate of $f_{\mathrm {s}}$ = 1 kHz results in a high sample density along the urethra which is beneficial for e.g. noise reduction. For signal reconstruction, data need to be downsampled in order to keep memory requirements and computation time within reasonable bounds. In order to avoid aliasing through downsampling, pressure data need to be lowpass filtered. The cutoff frequency depends on the retraction speed, catheter rotation during retraction, and desired resolution of the reconstructed image. As data are processed offline, they can be filtered forward and backward in time yielding zero-phase filtering which does not compromise the true shape of the signal [19, 20].

If deconvolution is applied, the necessary cutoff-frequency can be even lower than the one required by downsampling. The urethral tissue itself acts as a lowpass filter, therefore it limits the bandwidth of the sphincter-induced signal that can be registered by the catheter. This is discussed in detail in the section on deconvolution.

#### Signal reconstruction

As the catheter is retracted through the urethra, data are sampled at fixed time intervals and a sample pattern is generated on its surface. We assume that the samples lie on a cylindrical surface which can be mapped onto a two-dimensional plane with the coordinates $-\pi \cdot r_{\mathrm {c}}\le t<\pi \cdot r_{\mathrm {c}}$ with the catheter’s radius $r_{\mathrm {c}}$ and *s* (coordinate along urethra). The sensor trajectories and hence the pressure samples are mapped onto that surface (Fig. 4a). Before mapping pressure data onto that surface, they are zeroed with respect to vesicular pressure and cropped axially (along *s*) to the region of interest of length *l* which is usually the functional profile length at rest (FPL). Each sample location along *s* is calculated from the initial catheter insertion depth, position information of the puller device, and the axial sensor offset. Sample locations along $\theta$ are obtained by calculating the catheter orientation and compensating for the angular sensor offsets.

Additionally, the catheter’s inclination with respect to the horizontal plane is calculated from the acceleration data. The inclination data can be combined with the position data from the puller device to obtain the urethra’s geometry in the sagittal plane. To this end, starting from some point (e.g. the origin) the next point is calculated using the catheter’s inclination angle and the distance covered to obtain the next point and so forth.

Any signal reconstruction algorithm needs to be suitable for the spatial sample distribution it is applied to. If the catheter is retracted without rotation along its longitudinal axis the sample distribution is a regular rectangular grid and signal reconstruction is straightforward. Unfortunately, the catheter usually rotates on its own during retraction resulting in a nonuniform sample distribution due to the axial gaps between the sensors [11]. Reconstructing a multidimensional signal from nonuniform samples is not a trivial task. However, the high sampling rate along the sensor trajectories enables the generation of a sampling lattice as defined by Butzer and Hinsen [21, 22] through downsampling. This sampling approach (Fig. 4b) requires that all samples lie on straight lines parallel to the *v*-axis. Yet, those lines need not be equally spaced and the sample points on each line need not be uniformly distributed.

After mapping the sensor trajectories onto a two-dimensional plane (Fig. 4a), the lattice (Fig. 4c) is generated as follows (for details see [11]):

1. Expand the original sampling pattern periodically across left and right boundary so that a windowing function can be applied.
2. Calculate the mean trajectory angle $\tau$ to obtain *u* parallel to the mean trajectory and *v* perpendicular to *u*.
3. Generate lines parallel to *v*. Intervals between lines should be chosen small enough to satisfy the Whittaker-Kotel’nikov-Shannon sampling theorem in *s*-direction. The line intervals thereby determine the cutoff-frequency for the lowpass filter used to preprocess the pressure data.
4. Find samples on each trajectory closest to the intersection with each *v* -parallel line.
5. Resulting samples (orange dots) are placed at the intersections of *v*-parallel lines and trajectories with the value obtained in step 4.

The fourth step necessitates a high sampling frequency $f_{\mathrm {s}}$. Generally, pressure samples will not lie on the *v*-parallel lines, therefore violating the requirements for a Butzer and Hinsen sampling lattice in a strict sense. However, the high sampling rate in combination with slow retraction speed results in a very small sample distance of only a few micrometers along *u*. Therefore, the error through nearest-neighbor interpolation in step four is negligible compared to other measurement errors.

The Butzer and Hinsen sampling lattice allows for a separable reconstruction process. This means that first one-dimensional functions along *v* are reconstructed and then the final reconstructed points are calculated by applying one-dimensional reconstruction along *s*. This has the additional advantage that different composing functions can be used along both coordinates to suit their respective dynamics and boundary conditions. Reconstruction of the pressure distribution on the urethra’s inside is carried out in four steps [11]:

*Step 1:* Create Butzer-Hinsen-like sampling lattice through downsampling.

*Step 2:* Define points for signal reconstruction (green + in Fig. 4a, c). The points lie on a regular rectangular grid on lines parallel to *s* (red dashed lines). The intersection of the *s*-parallel with the *v*-parallel lines are the interpolation points used in the one-dimensional reconstruction along the *v*-parallel lines yielding virtual samples for the next reconstruction step along *s* (blue x).

*Step 3:* Calculate virtual samples (blue x in Fig. 4c) using the reconstruction equation along *v* from Margolis and Eldar [23] for periodic nonuniform sample distribution.

*Step 4:* Reconstruct pressure distribution at predefined points (green + in Fig. 4a, c) from virtual samples using the algorithm from Grishin and Strohmer [24], where symmetric rather than periodic boundary conditions are enforced.

The sample distribution has a significant influence on reconstruction stability, that is how small disturbances in the samples affect the reconstruction error. Generally, with increasing nonuniformity of the sample distribution the reconstruction stability decreases. In HD-UPP, reconstruction stability depends on sensor trajectory spacing which itself depends on catheter rotation and sensor placement on the catheter. It is shown in [11] that reconstruction stability quickly deteriorates as the catheter starts to rotate during retraction. On the other hand, catheter rotation increases angular resolution as sample density increases along the *v*-coordinate. Therefore, in future applications catheter rotation needs to be tightly controlled by the puller device and sensor positions can be optimized to guarantee optimal stability at a defined rotation velocity.

#### Deconvolution

Signal reconstruction from the previous step yields a pressure image on the urethra’s inside. The pressure distribution is mostly generated by the urinary sphincter which is separated from the catheter by urethral tissue. Therefore, the pressure distribution exerted by the urinary sphincter on the urethra’s outside is somewhat different from the one measured on the inside as pressure transmission through an elastic medium distorts it. Currently, SUI is surgically treated irrespective of the individual site of sphincter deficiency. Providing the physician with the precise location of sphincter deficiency could enable the development of focal therapy approaches for potentially better results and quicker recovery for the patient.

Reconstructing the pressure distribution on the urethra’s outside from known boundary conditions on its inside is an inverse problem. As the previous signal processing step yields a high resolution pressure image and pressure transmission through a linear elastic medium can be mathematically be described as a convolution [25], we propose to use deconvolution to solve the inverse problem.

The urethra’s pressure transmission properties are mathematically described in this approach through its convolution kernel or point-spread-function (PSF). As the actual pressure distribution around the urethra cannot be measured, the PSF needs to be identified through a finite-element (FE) model. A FE-model for the urethra and a suitable method to identify pressure transmission PSFs was introduced in [12] and the suitability of three different parametric PSFs was investigated. The model used to identify the PSFs was a linear-elastic isotropic cylindrical tube with the inner diameter $d= 3$ mm, outer diameter $D=4$ mm, Young’s Modulus $E= 3000$ MPa and Poisson’s ratio $\nu =0.48$. The forward problem was solved by applying 15 different pressure distributions on the outside in Ansys^®^ 14.5.7 and calculating the reaction on the inside. Both loads and reactions were imported into Matlab where the PSF parameters were identified by solving a least-squares optimization problem [12].

However, the identified PSF is only valid for a specific model parameter set. If any model parameter needs to be changed, the entire FE-model has to be rebuilt, the simulations re-run, and a new PSF shape parameter set identified which is a very time consuming task. We therefore generated a large dataset for different model parameters in order to parametrize the PSF shape parameters depending on model parameters. The inner diameter *d* remained constant as it corresponds to the catheter’s diameter. Forward simulations with 15 different load sets used in [12] were run for every combination of the parameter sets $D= \{4,4.5,5,6,7\}{\mathrm{mm}}$, $E=\{3.75,5,70,2000,3000,4000\} {\mathrm{MPa}}$, and $\nu =\{0.4,0.45,0.48,0.49\}$.

The resulting 1800 load and reaction sets (15 load sets $\times$ 5 diameters $\times$ 6 Young’s Moduli $\times$ 4 Poisson’s ratios) were used to identify the shape parameters of the truncated quadratic PSF

1 $$\begin{aligned} \tilde{w}(\bar{t},\bar{s})&= \frac{a_1^2\cdot a_2^2\cdot \left( a_1^2-a_3\cdot \bar{t}^2\right) \cdot \left( a_2^2+a_4\cdot \bar{s}^2\right) }{\left( \bar{t}^2+a_1^2\right) ^2\cdot \left( \bar{s}^2+a_2^2\right) ^2}+\alpha , \end{aligned}$$

presented in [12] with the tangential coordinate $\bar{t}$, the axial coordinate $\bar{s}$ and the continuous PSF $\tilde{w}$ with the shape parameters $\varvec{a}$ and the offset parameter $\alpha$. The center of the PSF is located at $(\bar{t},\bar{s})=(0,0)$ and it is defined on the domain $\bar{t}\in \left[ -\bar{t}_{\mathrm {max}},\bar{t}_{\mathrm {max}}\right]$, $\bar{s}\in \left[ -\bar{s}_{\mathrm {max}},\bar{s}_{\mathrm {max}}\right]$ where

2 $$\begin{aligned} \bar{s}_{\mathrm {max}}&= D-d\end{aligned}$$

3 $$\begin{aligned} \bar{t}_{\mathrm {max}}&= {\left\{ \begin{array}{ll} \bar{s}_{\mathrm {max}} &{} \quad \text {if}\; \bar{s}_{\mathrm {max}} \ge 2\,{\mathrm{mm}}\\ 2 \,\mathrm{mm} &{} \quad \text {otherwise.} \end{array}\right. } \end{aligned}$$

Scaling of the domain size is necessary to account for increasing PSF bluntness with increasing tissue thickness. As the PSF is applied to discrete data, it is discretized as well yielding the discrete PSF represented by the Matrix $\varvec{W}$ with the entries

4 $$\begin{aligned} w(\bar{t}_j,\bar{s}_k) = \beta \cdot \frac{\tilde{w}(\bar{t}_j,\bar{s}_k)}{\sum _j\sum _k \tilde{w}(\bar{t}_j,\bar{s}_k)}. \end{aligned}$$

and the scaling parameter $\beta$. For deconvolution we use the algorithm introduced by Levin et al. [26, 27] in the frequency domain.

**Table 1:** Functions to approximate PSF shape parameters

PSF shape parameter	Depends on model parameters	Approximation
$a_1$	*D*	Polynomial (degree 1)
$a_2$	*D*, $\nu$	Bilinear interpolation
$a_3$	*D*, $\nu$	Bilinear interpolation
$a_4$	*D*	Cubic interpolation
$\alpha$	*D*	Polynomial (degree 4)
$\beta$	*D*, $\nu$	Rational polynomial along *D* (degree 2/2), linear interpolation along $\nu$

The shape parameters strongly depend on *D* but appear invariant with respect to *E* (see Additional file 1). Therefore, the shape parameters are plotted depending only on *D* and $\nu$ in Fig. 5 (gray transparent surface). We additionally observe that $a_1$, $a_4$, and $\alpha$ are invariant with respect to $\nu$ as well. Table 1 shows model parameter dependencies of the shape parameters and the interpolation functions used to calculate values between the identified data points. These are valid for $D\in {[4,7]}\,{\mathrm{mm}}$ and $\nu \in \left[ 0.45,0.49\right]$. Due to the complex shape of the parameter surface of shape parameters $a_{2,3,4}$ (Fig. 5b–d) we use bilinear interpolation along $D,\nu$ for $a_{2,3}$ and cubic interpolation along *D* for $a_4$. Parameters $a_1$ and $\alpha$ are approximated by polynomials along *D* of degree one and four, respectively. Finally, $\bar{\beta }$ is interpolated by rational polynomials with the polynomial parameters $\varvec{c}$

5 $$\begin{aligned} \bar{\beta }_i(D,\nu _i) = \frac{c_{1,i}\cdot D^2+c_{2,i}\cdot D+c_{3,i}}{D^2+c_{4,i}\cdot D+c_{5,i}} \end{aligned}$$

along *D* for $\nu _i=\{0.4,0.45,0.48,0.49\}$ and $\beta (D,\nu )$ is obtained by linearly interpolating between two adjacent $\bar{\beta }_i(D,\nu _i)$. The results are displayed in Fig. 5, where the gray transparent surface representing the identified PSF shape parameters and the colored interpolating surfaces match very well.

As elastic tissue has a smoothing (that is lowpass filter) effect on pressure transmission, the solution to the inverse problem causes sharpening of the input pressure image, thereby amplifying disturbances in high frequencies as well. To mitigate this effect, input data can be lowpass filtered before applying signal reconstruction and deconvolution. In order to determine a suitable cutoff-frequency, we analyze the PSF as it represents the impulse response of a linear filter. We apply the 2D-Fourier-Transform

6 $$\begin{aligned} W(\omega _{\mathrm {t}},\omega _{\mathrm {s}}) = \int _{-\infty }^{\infty }\int _{-\infty }^{\infty } \tilde{w}(\bar{t},\bar{s})\cdot \exp \left( -2 \pi j\cdot \left( \omega _{\mathrm {t}}\cdot \bar{t}+\omega _{\mathrm {s}}\cdot \bar{s}\right) \right) \,\mathrm {d}\bar{t}\,\mathrm {d}\bar{s} \end{aligned}$$

to Eq. (1). Under the assumption of real parameters and positive $a_{1,2}$ we obtain the continuous 2D-Fourier-transformed PSF

7 $$\begin{aligned} W(\omega _{\mathrm {t}},\omega _{\mathrm {s}}) =\;&\alpha \cdot \delta (\omega _{\mathrm {t}},\omega _{\mathrm {s}})-\frac{a_1\cdot a_2\cdot \pi ^2}{4}\cdot \exp \left( -2\pi \cdot \left( a_1\cdot \omega _{\mathrm {t}}+a_2\cdot \omega _{\mathrm {s}}\right) \right) \cdot \nonumber \\&\left( a_3+2\pi \cdot a_1\cdot \omega _{\mathrm {t}} \cdot \left( 1+a_3\right) -1 \right) \cdot \nonumber \\&\left( a_4-2\pi \cdot a_2\cdot \omega _{\mathrm {s}}\cdot \left( a_4-1 \right) +1\right) \end{aligned}$$

where $$\omega _{\mathrm {t}},\omega _{\mathrm {s}}\ge 0$$ and

8 $$\begin{aligned} \delta (\omega _{\mathrm {t}},\omega _{\mathrm {s}}) = {\left\{ \begin{array}{ll} 1 &{} \quad \text {if } \omega _{\mathrm {t}}=\omega _{\mathrm {s}}=0\\ 0 &{} \quad \text {otherwise.} \end{array}\right. } \end{aligned}$$

Note that due to the definition of the Fourier-Transform in Eq. (6), $\omega _{\mathrm {t,s}}$ are spatial frequencies with the unit ${1}/{\mathrm{mm}}$. Thanks to the linearity of the Fourier-Transform we can ignore scaling of $w(\bar{t},\bar{s})$ in Eq. (4) as it only scales $W(\omega _{\mathrm {t}},\omega _{\mathrm {s}})$ as well.

We define the PSF cutoff-frequency at $-6\; {\mathrm{dB}}$, that is from whereon less than a quarter of the signal power is transmitted. As the PSF is a two-dimensional function, the cutoff-frequency is a line in the $\omega _{\mathrm {t}},\omega _{\mathrm {s}}$-plane. The results for different *D* are shown in Fig. 6. As expected, the cutoff-frequency lines move towards lower frequencies with increasing *D*. In order to design a lowpass filter, a frequency on each line needs to be selected. We choose the lowest frequency on each line which is located on the $\omega _{\mathrm {s}}$-axis. This is convenient, as data are approximately recorded and filtered along the *s*-coordinate as well. Additionally, the cutoff-frequency appears to be invariant with respect to $\nu$. Therefore, we fit a power model

9 $$\begin{aligned} f_{\mathrm {cut}}(D) = c_1\cdot D^{c_2}+c_3 \end{aligned}$$

with the parameters $\varvec{c}$ to the identified data points along *D*, which is depicted in Fig. 7. The cutoff-frequency can thereby be calculated from the algorithm parameter *D* and compared to the one depending on the signal reconstruction properties. The lower one is then applied to pressure data before signal reconstruction (cf. section on lowpass filtering).

### Experimental setup

For this work, female Göttingen minipigs (Ellegaard, Dalmose, Denmark) between 12 and 18 months old were used for HD-UPP experiments. This study was part of the scientific program of the “Klinische Forschergruppe 273” (KFO273). The local ethics committee^1^ approved the experiments (reference number CU 1/12) and a veterinary license has been obtained. The minipigs were sedated using a combination of Atropin (0.1 %, 0.05 ml/kg), Stresnil^®^ (Azaperon 4 %, 0.1 ml/kg), Ketamin (10 %, 0.14 ml/kg), and Midazolam (0.5 %, 0.04 ml/kg), and without muscle relaxation scouring. All efforts were made to minimize suffering.

The microtip catheter was inserted through a cystoscope and then the cystoscope was retracted. Data recording was started simultaneously with catheter retraction and the retraction speed $v_{\mathrm {r}}$ was set between 1 and 2 mm/s. Retraction was stopped as soon as pressure sensors exited the urethra. Note that different retraction speeds do not affect signal reconstruction in HD-UPP, as the catheter’s retraction distance is simultaneously recorded to pressure and inclination sensor data. Therefore, pressure images and their features obtained at different retraction speeds are comparable. The catheter’s rotation was measured by using the inclination sensor data to calculate its orientation at all times during the measurement.

As shown in [14], the HD-UPP catheter delivers plausible results in when compared to an air-charged catheter as gold standard in agreement with clinical studies on the comparison of microtip and air-charged catheters. To underline the validity of HD-UPP data, we include the urethral pressure traces recorded directly before the HD-UPP data using an air-charged catheter (T-DOC-7FD, T-DOC Company, Wilmington, USA) connected to an Aquarius TT^™^ data acquisition unit (Laborie, Mississauga, Canada) in Additional file 2.

## Results

Experimental results from data obtained from three female minipigs are shown in Figs. 8, 9, 10 (one figure for each minipig). For comparison, we show results obtained without and with deconvolution in the processing work flow in the left and right columns, respectively. PSFs were calculated for $\nu =0.48$, which is suitable for elastic tissue, and $D=7 \, {\mathrm{mm}}$, as the effect of pressure transmission through tissue increases with its thickness. For lower values of *D* the PSF’s cutoff-frequency increases as its bluntness decreases and converges towards an impulse. Therefore, both pressure images inside and outside in the deconvolution work flow converge towards the pressure image inside (without deconvolution) as *D* decreases.

In order to assess the plausibility of the HD-UPP pressure traces in Figs. 8, 9, 10a they can be compared to their respective pressure traces obtained with the air-charged catheter in Additional file 2. As the air-charged catheter was retracted at $v_{\mathrm {r}}= 1 \, {\mathrm{mm}}/{\mathrm{s}}$, the time scale corresponds to a distance scale in millimeters. As expected, the overall shape of the pressure traces is very similar as well as the length of the pressure profile. Generally, the pressures recorded by the air-charged catheter are higher than the ones recorded by the HD-UPP catheter. Those findings are in agreement with clinical studies on the comparison of microtip and air-charged catheters [6, 28], showing the validity of the recorded HD-UPP data.

Sub-figures 8, 9, 10a show the lowpass filtered pressure traces from the eight urethral pressure sensors along the urethra. The cutoff-frequency was defined by the downsampling step for signal reconstruction. Additionally, artifact elimination was applied to the signals. From the preprocessed signals displayed in Sub-figures 8, 9, 10a, the pressure images on the urethra’s inside (Sub-figures 8, 9, 10b) were reconstructed. The pressure image from Sub-figures 8, 9, 10b was then mapped onto a cylindrical surface along the urethra’s geometry in the sagittal plane (*xz*) in Sub-figures 8, 9, 10c. Sub-figures 8, 9, 10d depict the pressure image on the urethra’s inside reconstructed from pressure traces lowpass filtered according to the PSF’s cutoff-frequency. Deconvolution was applied to the images in Sub-figures 8, 9, 10d and the results are shown in Sub-figures 8, 9, 10e. For easier comparison of the differences between Sub-figures 8, 9, 10d, e, we subtract the pressure images in 8, 9, 10e from the ones in 8, 9, 10d and show the results in Sub-figures 8, 9, 10f. We additionally provide 3D-PDF files of the mapped pressure image on the urethral geometry in Additional files 3, 4, and 5 for minipigs 1, 2, and 3 respectively.

There is a considerable variation in the results between minipigs 1–3 in terms of pressure distribution, maximum pressure, and urethral geometry. The FPL of the three minipigs ranges from 70 mm to 85 mm and the maximum pressure inside from $117\,{\mathrm{cm}\,{\mathrm{H}_2}{\mathrm{O}}}$ to $129\, {\mathrm{cm}\,{\mathrm{H}_2}{\mathrm{O}}}$. The maximum pressure is located between approximately 50 mm and 60 mm from the urethra’s exit. Pressure levels are lower in the deconvolved images, as the cylindrical area on the urethra’s outside is larger than on its inside, which causes pressure amplification from outside to inside.

Deconvolution generally sharpens the pressure image somewhat and can also lead to changes in the pressure distribution. In minipig 1, pressure peaks apparent on the inside are reduced on the outside (Fig. 8d–f, details x and y). Additionally, the smoothly curved distal edge of the high pressure zone on the inside exhibits a dent and thereby a slight weak spot on the outside (Fig. 8d–f, detail z). In minipig 2, pressure peaks appear more pronounced and additional contours become visible (Fig. 9d–f, details x, y, and z). On the contrary, deconvolution hardly amplifies any details when applied to data from minipig 3 (Fig. 10d–f) resulting in a rather smooth looking pressure difference image (Fig. 10f).

The benefits of signal reconstruction are illustrated in Fig. 11 through simulation results. Instead of implementing a comparatively elaborate signal reconstruction algorithm, the pressure distribution could be approximated by performing Delaunay-triangulation on the pressure samples and bilinear interpolation along the resulting triangles. This simple approach can lead to inaccuracies in the reconstructed pressure image. The left and right columns show the results for two different test functions a and b displayed in the top row. The functions are sampled at the locations indicated by colored dots, each color representing a different pressure sensor. The sample distribution was simulated to match that of the microtip catheter for straight retraction (left column) and retraction with slight catheter rotation at $7 {\,\mathrm{deg}}/{\mathrm{mm}}$ (right column). Note the nonuniform trajectory spacing due to catheter rotation. The test functions were reconstructed using Delaunay-triangulation and bilinear interpolation (middle row) and our signal reconstruction algorithm (bottom row).

Results for evaluating the effectiveness of artifact elimination are shown in Fig. 12. The Sub-figures depict cropped and enlarged sections in the high pressure zones of the pressure images inside from minipigs 1–3 (Figs. 8, 9, 10b) from top to bottom, respectively. In the left column Sub-figures were generated without artifact elimination and vertical ripple patterns from vascular pulsation are clearly visible in the high pressure areas. The images in the right column were generated using PCAD and removing the first two principal components from the pressure data before applying signal reconstruction. The vertical ripple patterns are successfully removed while other fine details are well preserved.

## Discussion

The results show that a high detail pressure image can be reconstructed on the urethra’s inside, which allows for spatial data location and a significantly more intuitive interpretation compared to looking at pressure traces. In comparison, bilinear interpolation cannot provide the same level of accuracy. One self-explanatory reason is that pressure maxima and minima are always located at sample locations (colored dotted lines in Fig. 11) when using bilinear interpolation. This can lead to mislocation of pressure maxima and minima and underestimation of their size. Under favorable conditions (i.e. sensors pass exactly over maxima and minima) the deviation from the original function is small (Fig. 11c). If the sample distribution is not as favorable, the reconstruction error can become significant (Fig. 11d). Here, maxima and minima are both mislocated, distorted along the trajectories, and their magnitude is misestimated. On the contrary, our signal reconstruction algorithm manages to recover the original functions accurately (Fig. 11e and f). The signal reconstruction algorithm can recover the correct location and magnitude of maxima and minima even if sensors do not pass exactly over them, due to appropriate assumptions on bandwidth of the original function and boundary conditions (for details see [11]). Applying deconvolution to a pressure image amplifies errors even further. Therefore, implementing a sophisticated signal reconstruction algorithm instead of simple bilinear interpolation is important to provide the physician with the most accurate image possible for diagnosis.

There is a visible difference between the pressure images inside (Sub-figures 8, 9, 10d) and the deconvolved images (Sub-figures 8, 9, 10e and f). The pressure image inside with the cutoff-frequency limited by the downsampling step (Sub-figures 8, 9, 10b) is more detailed due to the higher cutoff-frequency. In minipigs 1 and 2 changes in the pressure distribution from the inside (Sub-figures 8d and 9d) to the deconvolved image outside (Sub-figures 8e and 9e) can be observed. These differences are unlikely to be artifacts caused by deconvolution, as sometimes there are hardly any changes to the details in the pressure distribution at all (minipig 3, Fig. 10). Additionally, the accuracy and robustness of the deconvolution approach has been verified through simulations in [12]. However, in the current approach it is assumed that all model parameters do not change along the urethra’s axial or angular coordinate. While it is possible to use a coordinate dependent PSF in deconvolution, the problem of determining the coordinate dependent model parameters for any given individual remains. However, according to our simulation results, the PSF appears to be invariant with respect to the Young’s Modulus and therefore its variations in the urethral tissue can be ignored. The Poisson ratio only slightly affects the PSF’s shape and variations in $\nu$ hardly have a visible effect on the results. Moreover, human tissue is known to be almost incompressible, we therefore do not expect great variations within the urethral tissue. Tissue thickness has a significant influence on the PSF’s shape. In practice, this parameter varies along the urethra and between individuals. Moreover, the different tissue layers in and around the urethra are not separated by a sharp boundary [29–31] adding further variance to *D*. A possible remedy is to reconstruct the pressure image at varying tissue thicknesses and thereby check whether features of interest appear consistently across the layers.

The differences between pressure images inside and outside aside from pressure scaling are generally rather small. This appears reasonable, as the modeled tissue thickness was only $1.5 {\,\mathrm{mm}}$ and any dramatic changes would most likely point to robustness problems of the deconvolution algorithm. Additionally, data were obtained from healthy minipigs, therefore there are no pathological conditions in the sphincter to detect. An additional use of modeling urethral tissue through a PSF is calculating the bandwidth of the signal that an anatomic structure (e.g. the external urethral sphincter) transmits onto the catheter. Thereby, higher frequency components in the pressure signal (from structures closer to the catheter) can be eliminated to provide a cleaner image for assessing the pressure exerted by the anatomic structure of interest. This could help to accurately pinpoint the location of sphincter deficiency for a more focused therapy approach.

Artifact elimination using PCAD is very effective at removing time-correlated artifacts from pressure data while preserving other details in the pressure images. This is a major advantage over bandstop or lowpass filters, which are effective at removing artifacts in their respective stop-band but eliminate all other features within it as well. Time-correlation based artifact elimination is another unique feature in HD-UPP, as it requires a high number of pressure sensors to work effectively.

An open question is which pressure image is more useful or accurate for diagnosing SUI, the detailed one on the inside or the deconvolved one on the outside. To answer this question, further research is needed with data from subjects suffering from sphincter deficiency. The deconvolution step itself is optional and its diagnostic use needs to be investigated further. However, the PSF-based pressure transmission model can still be useful for estimating a cutoff-frequency for the pressure transmission to obtain a cleaner pressure image on the urethra’s inside. Data from subjects with sphincter deficiency are also needed to assess the actual diagnostic benefits of high resolution pressure images. Especially angular fluctuations in the pressure distribution are often dismissed as artifacts. The high reproducibility of HD-UPP pressure images [14], however, strongly suggests that angular pressure fluctuations are of physiological rather than artificial nature. Currently, HD-UPP provides researchers with a set of tools to evaluate data under different assumptions, that is whether relevant diagnostic information is mostly contained in the pressure image inside, outside, or both. Additionally, the urethral geometry can be reconstructed in the sagittal plane and the pressure distribution mapped onto it and plotted three-dimensionally. Therewith, the effect of urethral curvature on the measured pressure distribution and related hypothesis can be investigated as well. Future (pre-)clinical studies will have to show if the added cost and complexity of HD-UPP compared to conventional systems results in corresponding diagnostic value.

## Conclusions

In conventional UPP low spatial pressure resolution, artifacts, lack of spatial data location, and unintuitive results presentation limit its diagnostic value. In order to address these problems, HD-UPP was developed employing new diagnostic hardware and signal processing algorithms. A novel microtip catheter measures urethral pressure with unprecedented spatial resolution. Additionally, an integrated inclination sensor in combination with position data from the puller device allows spatial pressure data location as well as calculating the urethra’s geometry in the sagittal plane. The signal processing steps presented in this work enhance the collected data to provide detailed and reliable results that can be intuitively interpreted by the physician.

Although surgical therapy of SUI to date focuses on general treatment irrespective of the individual site of sphincter deficiency, further treatment approaches based on focal therapy might be enabled by spatial definition of sphincter complex’s defect. Results from female minipigs show that a high resolution pressure image on the urethra’s inside can be successfully reconstructed and data spatially located. Additionally, the catheter’s sensor configuration facilitates removal of time-correlated artifacts without blurring other details in the image. Finally, the pressure distribution in the surrounding tissue can be calculated using deconvolution. The tissue is modeled as a PSF and its shape parameters depending on tissue model parameters are identified through simulations. This optional step enables analyzing the pressure distribution exerted by the sphincter onto the urethra which can deviate from the pressure distribution measured inside. Therefore, HD-UPP provides the physician with different signal processing tools. Each tool provides slightly different details and insights on the urethral pressure distribution. In order to compare their diagnostic value separately and combined, further studies need to be conducted.

In conclusion, HD-UPP overcomes many limitations of traditional UPP and can thereby provide new diagnostic value in urodynamics. It could provide tools in future studies to better discriminate different forms of SUI (such as intrinsic sphincter insufficiency or urethral hypermobility) and support the selection of the optimal treatment.

## Additional files

10.1186/s12938-016-0145-6 Identified PSF shape parameters. PSF shape parameters depending on *D*, $\nu$, and *E*.
10.1186/s12938-016-0145-6 Minipig UPPs from an air-charged catheter. Pressure profiles (urethral pressure) obtained with an air-charged catheter. Top to bottom: Minipig 1–3, respectively.
10.1186/s12938-016-0145-6 3D results (minipig 1). Pressure image inside (minipig 1) mapped onto urethra geometry in the sagittal plane. Note that in order to view 3D-PDF files, the Adobe $^{\textregistered }$ Acrobat Reader $^{\textregistered }$ is required (https://get.adobe.com/reader/). Third-party PDF viewers generally do not display them correctly.
10.1186/s12938-016-0145-6 3D results (minipig 2). Pressure image inside (minipig 2) mapped onto urethra geometry in the sagittal plane. Note that in order to view 3D-PDF files, the Adobe $^{\textregistered }$ Acrobat Reader $^{\textregistered }$ is required (https://get.adobe.com/reader/). Third-party PDF viewers generally do not display them correctly.
10.1186/s12938-016-0145-6 3D results (minipig 3). Pressure image inside (minipig 3) mapped onto urethra geometry in the sagittal plane. Note that in order to view 3D-PDF files, the Adobe $^{\textregistered }$ Acrobat Reader $^{\textregistered }$ is required (https://get.adobe.com/reader/). Third-party PDF viewers generally do not display them correctly.

## Footnotes

1 Regierungspräsidium Tübingen, P.O. Box 26 66, 72016 Tübingen, Germany. File number 35/9185.81-2.
