# Supplementary figures and images for: Signal processing in urodynamics: towards high definition urethral pressure profilometry
Source: Biomed Eng Online. 2016 Mar 22;15:31. doi: 10.1186/s12938-016-0145-6 (PMC4802619; doi:10.1186/s12938-016-0145-6)

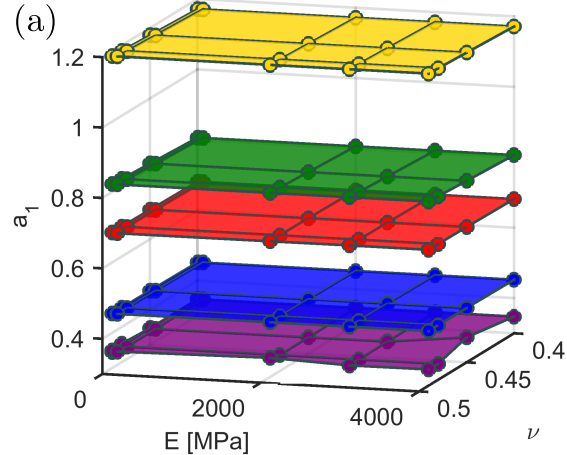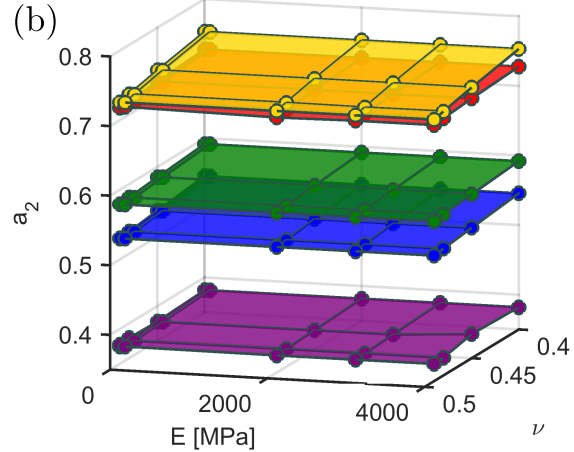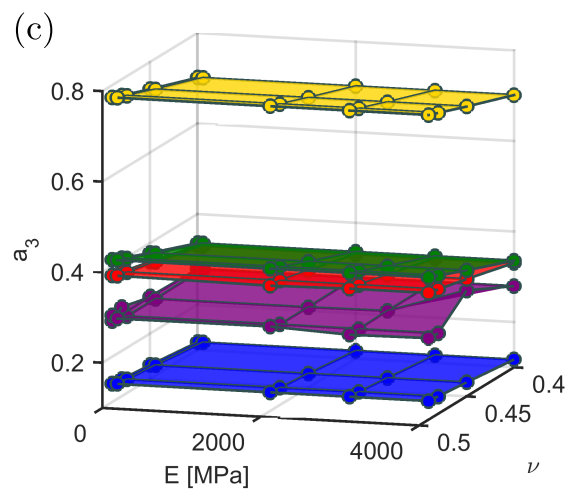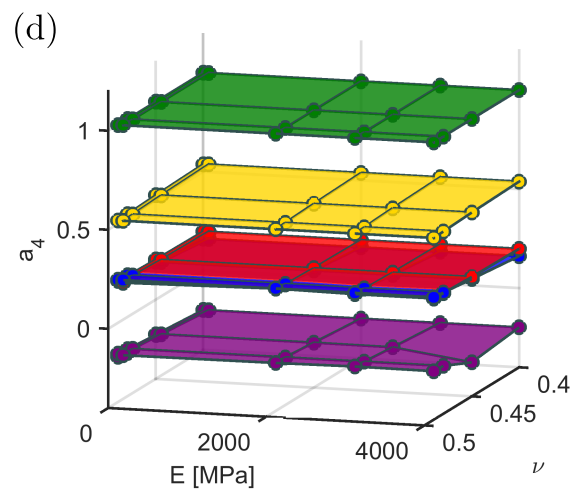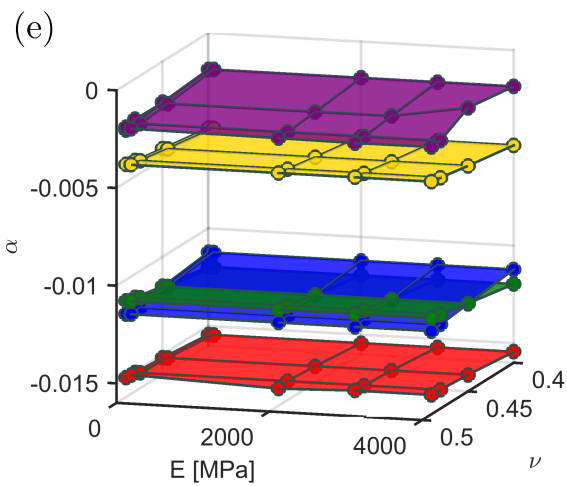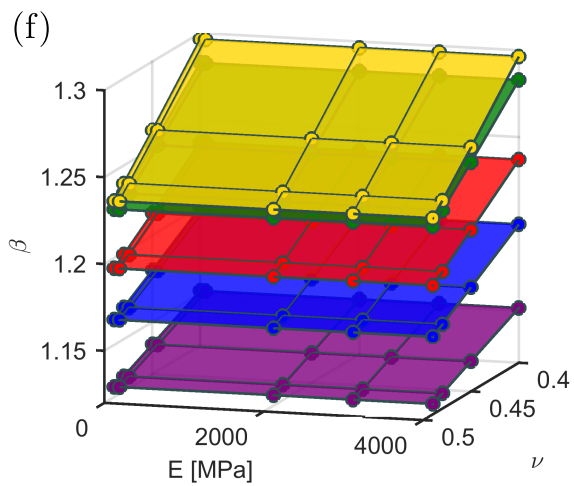

Supplement: Supplementary file 1 — 10.1186/s12938-016-0145-6 Identified PSF shape parameters. PSF shape parameters depending on D, $\nu$ν, and E. [file 12938_2016_145_MOESM1_ESM.pdf]

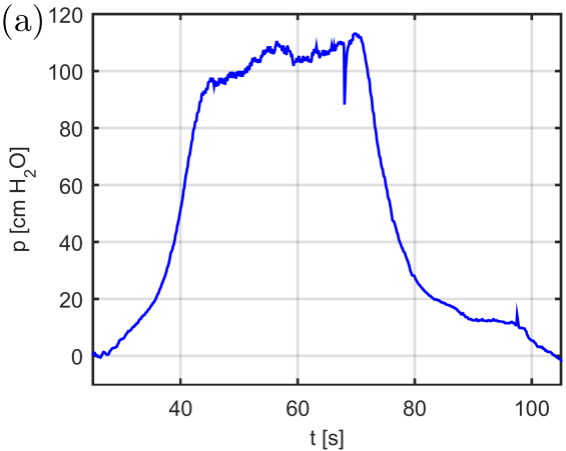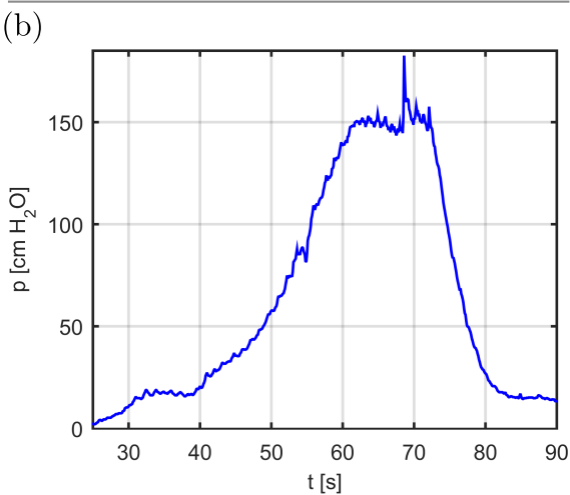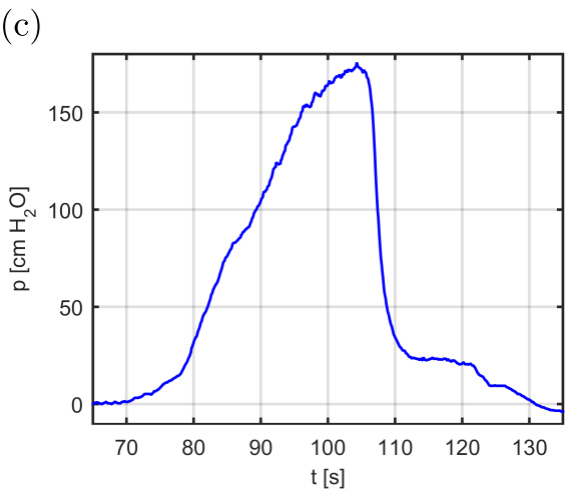

Supplement: Supplementary file 2 — 10.1186/s12938-016-0145-6 Minipig UPPs from an air-charged catheter. Pressure profiles (urethral pressure) obtained with an air-charged catheter. Top to bottom: Minipig 1–3, respectively. [file 12938_2016_145_MOESM2_ESM.pdf]

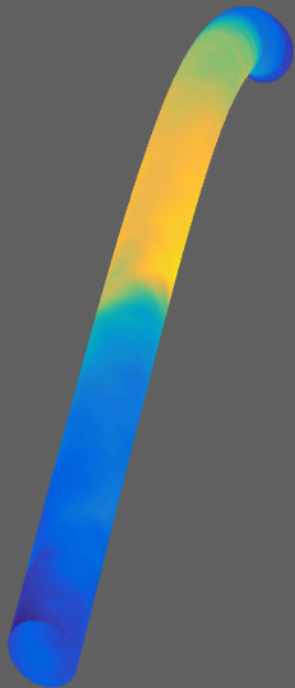

Supplement: Supplementary file 3 — 10.1186/s12938-016-0145-6 3D results (minipig 1). Pressure image inside (minipig 1) mapped onto urethra geometry in the sagittal plane. Note that in order to view 3D-PDF files, the Adobe $^{\textregistered }$® Acrobat Reader $^{\textregistered }$® is required (https://get.adobe.com/reader/). Third-party PDF viewers generally do not display them correctly. [file 12938_2016_145_MOESM3_ESM.pdf]

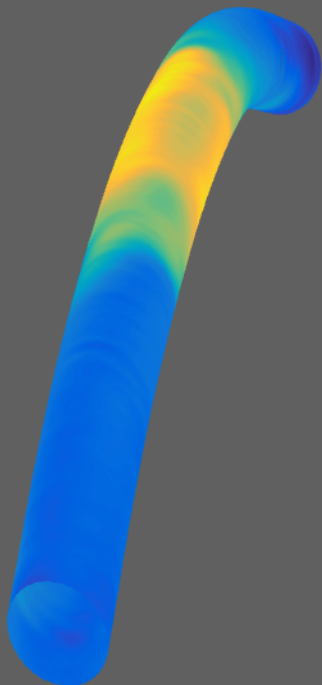

Supplement: Supplementary file 4 — 10.1186/s12938-016-0145-6 3D results (minipig 2). Pressure image inside (minipig 2) mapped onto urethra geometry in the sagittal plane. Note that in order to view 3D-PDF files, the Adobe $^{\textregistered }$® Acrobat Reader $^{\textregistered }$® is required (https://get.adobe.com/reader/). Third-party PDF viewers generally do not display them correctly. [file 12938_2016_145_MOESM4_ESM.pdf]

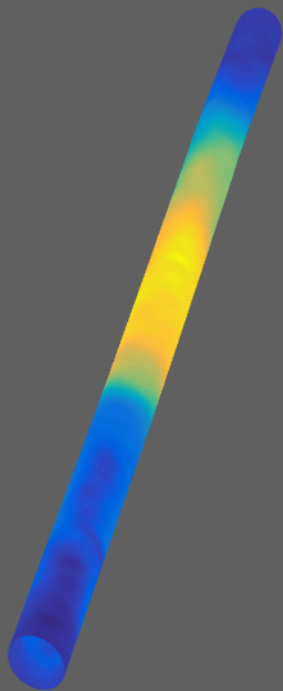

Supplement: Supplementary file 5 — 10.1186/s12938-016-0145-6 3D results (minipig 3). Pressure image inside (minipig 3) mapped onto urethra geometry in the sagittal plane. Note that in order to view 3D-PDF files, the Adobe $^{\textregistered }$® Acrobat Reader $^{\textregistered }$® is required (https://get.adobe.com/reader/). Third-party PDF viewers generally do not display them correctly. [file 12938_2016_145_MOESM5_ESM.pdf]
